# Regulatory T cells in cardiac allograft vasculopathy: from mechanistic insights to clinical tolerance

**DOI:** 10.3389/fimmu.2026.1893663

**Published:** 2026-08-05

**Authors:** Buyan Li, Yujing Su, Yanglin Hao, Weicong Ye, Song Wang, Xiaohan Li, Ran Li, Kexiao Zheng, Zifeng Zou, Yilong Li, Zetong Tao, Jie Wu, Xi Zhang, Jiahong Xia

**Affiliations:** 1 Department of Cardiovascular Surgery, Union Hospital, Tongji Medical College, Huazhong University of Science and Technology, Wuhan, China; 2 Tongji Medical College, Huazhong University of Science and Technology, Wuhan, China; 3 Center for Translational Medicine, Union Hospital, Tongji Medical College, Huazhong University of Science and Technology, Wuhan, China; 4 Institute of Translational Medicine, Tongji Medical College, Huazhong University of Science and Technology, Wuhan, China; 5 Key Laboratory of Organ Transplantation, Ministry of Education, Chinese Academy of Medical Sciences, Wuhan, China; 6 NHC Key Laboratory of Organ Transplantation, Chinese Academy of Medical Sciences, Wuhan, China; 7 Key Laboratory of Organ Transplantation, Chinese Academy of Medical Sciences, Wuhan, China

**Keywords:** cell- and tissue-based therapy, graft rejection, heart transplantation, immune tolerance, regulatory, T-lymphocytes

## Abstract

Cardiac allograft vasculopathy (CAV) is the primary impairment that influences the long-term prognosis of transplanted hearts. CAV is characterized by diffuse intimal hyperplasia of the coronary arteries, which is mediated by chronic inflammation, the alloimmune response, and vascular remodeling. Current immunosuppressive regimens effectively control acute rejection but have limited efficacy in preventing CAV and are associated with significant adverse effects upon long-term use. Regulatory T cells (Tregs) are essential for preserving immunological homeostasis and facilitating transplantation tolerance. They are pivotal in suppressing the activation of effector T cells (Teffs), modulating local inflammation, and postponing the progression of CAV. This review comprehensively elucidates the pathophysiology of and diagnostic advancements in CAV, emphasizes the phenotypic heterogeneity, immunosuppressive mechanisms, and protective role of Tregs in heart transplantation, and thoroughly discuss the interplay of PD-1/PD-L1, IL-33, IL-6, CTLA-4, fatty acid oxidation (FAO), and other signaling pathways in modulating Treg function and CAV pathogenesis. In terms of the translational medicine, the adoptive infusion of *in vitro*-expanded autologous Tregs has demonstrated the ability to postpone CAV in preclinical models; nonetheless, its clinical use is limited by cell stability, challenging preparation processes, and the lack of efficacy biomarkers. Therefore, this review seeks to establish a theoretical foundation and research viewpoint to comprehensively understand the immunological mechanisms of CAV and the advancement of novel Treg-targeted therapies.

## 1 Introduction

Heart transplantation is a viable intervention for individuals with end-stage heart failure, as it could successfully preserve the lives of numerous patients while markedly improving their quality of life and long-term survival rates (1). Cardiac allograft vasculopathy (CAV), the predominant type of chronic rejection, remains the primary barrier to improving the long-term survival of cardiac allografts and patient outcomes (2, 3). According to the International Society for Heart and Lung Transplantation (ISHLT) registry data report, the prevalence of CAV increases progressively with time after transplantation, affecting 8% of recipients at 1 year, 29% at 5 years, and up to 47% at 10 years (4). A recent national registry analysis also reported that the 5-year cumulative incidence of angiographic CAV reaches 33% among isolated heart transplant recipients (5), underscoring its persistent impact on allograft outcomes. CAV lesions are distinctive and progressive, marked by diffuse, concentric constriction of the graft coronary arteries, ultimately resulting in graft ischemia and dysfunction (6, 7). In contrast to that of conventional atherosclerosis, the pathogenesis of CAV is more intricate and involves donor-specific immune responses, inflammatory cascades, endothelial cell damage, the proliferation and migration of vascular smooth muscle cells (VSMCs), and the synergistic influence of nonimmune variables (8, 9). Consequently, a comprehensive understanding of the immunological mechanisms underlying CAV and the formulation of specific immunomodulation methods are crucial for extending the longevity of cardiac allografts.

While conventional immunosuppressive approaches have managed acute rejection after heart transplantation to some degree, the adverse effects associated with their prolonged use and their insufficient ability to prevent CAV have driven researchers to investigate more targeted and tolerance-promoting immunomodulatory strategies (1, 10). The pivotal function of regulatory immune cell subsets, particularly regulatory T cells (Tregs), in sustaining immunological homeostasis and fostering immune tolerance has garnered significant attention (11–14). Tregs are a subset of CD4^+^CD25^+^ T cells that express the transcription factor Foxp3. They impede the activation and proliferation of effector T cells (Teffs) through various mechanisms, thereby preserving immune homeostasis, preventing autoimmune diseases, and facilitating the acceptance of allogeneic grafts after organ transplantation (15–17). In the context of cardiac transplantation, Tregs are considered promising therapeutic targets for fostering graft tolerance and mitigating acute and chronic rejection (18–20). However, the precise mechanism of action of Tregs in the intricate pathophysiology of CAV, as well as the efficient use of Treg-guided therapy for the prevention and treatment of CAV, remain significant challenges in contemporary research.

This review is intended to systematically elucidate the immunopathological mechanisms of CAV, emphasizing the pivotal regulatory function of Tregs in the onset and progression of CAV and its associated molecular network. It also objectively assesses the research advancements and transformative potential of Treg-targeted immune intervention strategies for the prevention and treatment of CAV while anticipating the prospective development of a novel system for precise CAV prevention and treatment through the integration of multiomics analyses, metabolic immune regulation, and engineered therapeutic strategies.

## 2 Pathophysiological foundations and advancements in the diagnosis of CAV

The pathological characteristics of CAV include diffuse intimal thickening of the coronary artery of the graft, resulting from the proliferation and migration of smooth muscle cells and the accumulation of extracellular matrix, ultimately causing vascular stenosis, impaired blood flow, and subsequent myocardial ischemia, heart failure, and potentially sudden death (21, 22). In contrast to primary atherosclerotic lesions, CAV lesions are diffusely and concentrically distributed and primarily affect small and medium-sized arteries (23), hence complicating diagnosis and therapy.

### 2.1 Pathogenesis of CAV

**Figure 1:**
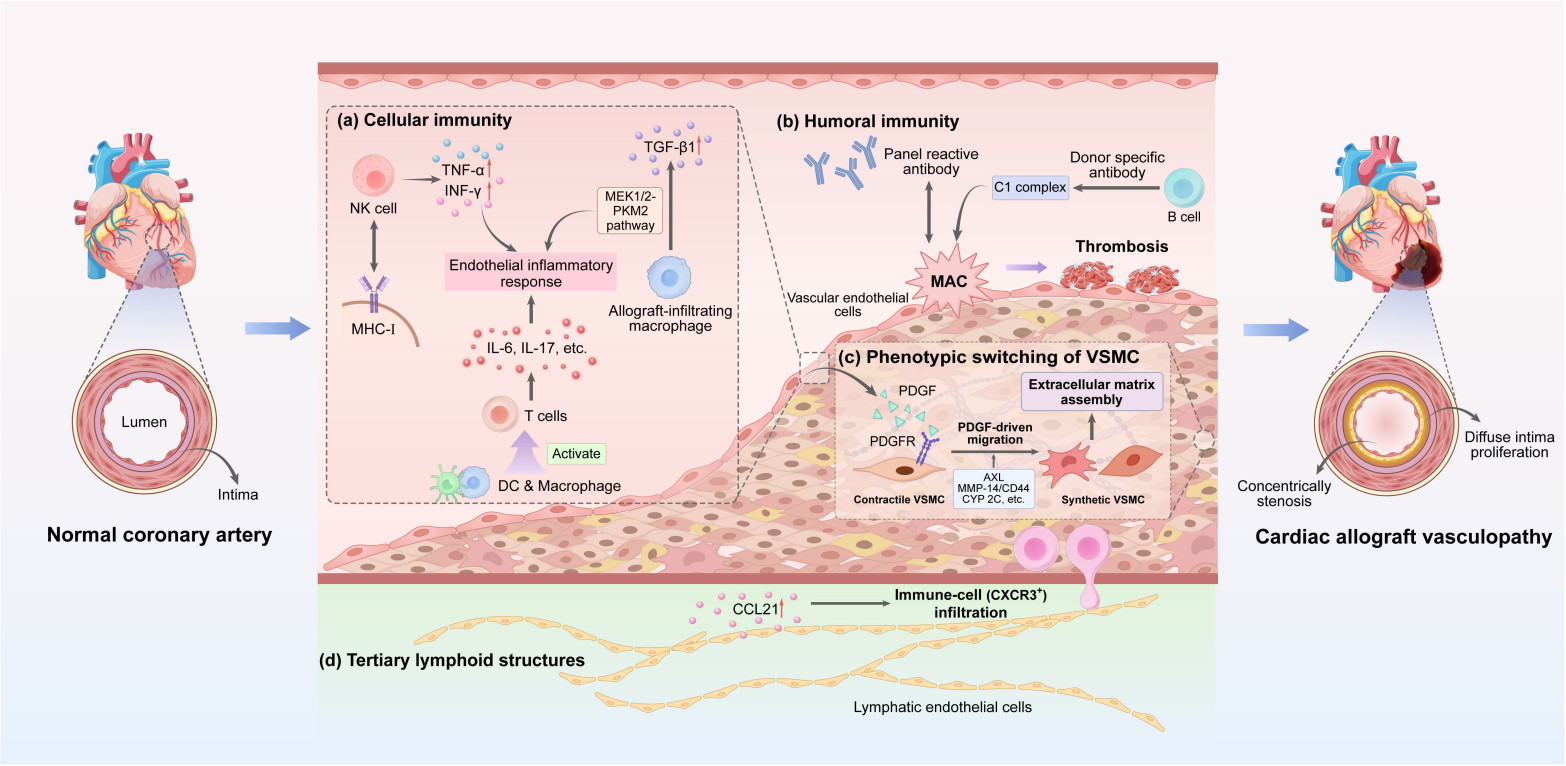
Pathogenesis of CAV: **(a)** Cellular immunity. Recipient NK cells recognize graft MHC-I and release TNF-α and IFN-γ, promoting endothelial inflammation. Activated dendritic cells and macrophages recruit T lymphocytes and produce cytokines such as IL-6, IL-17, etc. Allograft-infiltrating macrophages undergo metabolic reprogramming through the MEK1/2-PKM2 pathway, increasing TGF-β1 and sustaining inflammation. **(b)** Humoral immunity. B lymphocytes generate donor-specific antibodies and panel reactive antibodies that bind to the graft endothelium, which activate the classical complement pathway from C1 complex assembly to membrane attack complex (MAC) formation. This induces endothelial cytotoxicity, thrombosis, and exposure of cryptic epitopes, perpetuating immune-mediated vascular injury. **(c)** VSMC phenotypic switching. Inflammatory mediators such as PDGFs from inflammatory endothelium induce vascular smooth muscle cells to switch from contractile to synthetic phenotypes. This process involves AXL, MMP-14/CD44, and CYP2C signaling, leading to VSMC migration, proliferation, and extracellular matrix deposition in the intima. **(d)** Tertiary lymphoid structures. Lymphatic endothelial cells within the graft upregulate CCL21 expression, establishing chemotactic gradients that recruit CXCR3-expressing immune cells. This facilitates tertiary lymphoid structure formation, creating localized sites of sustained alloimmune activation that drive CAV progression.

The pathogenesis of CAV is multifaceted, encompasses various causes and stages and is characterized by the synergistic interactions of immunological and nonimmune elements (24). After heart transplantation, the alloimmune response serves as the primary mediator of CAV. T-cell activation and recruitment drive the cellular immune response against graft endothelial cells, contributing to vascular inflammation and endothelial injury (**Figure 1a**). While in the humoral immunity, donor-specific antibodies (DSAs) activate the classical complement cascade, leading to antibody-mediated endothelial injury (**Figure 1b**). The inflammatory microenvironment, driven by ischemia-reperfusion injury and innate immune effectors including macrophages and natural killer (NK) cells, promotes endothelial cell activation and secretion of proinflammatory cytokines (**Figure 1a**). Within this inflammatory milieu, VSMCs undergo phenotypic switching from a contractile to a synthetic state, migrate into the intima, and produce excessive extracellular matrix (**Figure 1c**). Tertiary lymphoid structure formation further amplifies the alloimmune response (**Figure 1d**), ultimately resulting in diffuse intimal hyperplasia and progressive luminal narrowing. The interplay among these mechanisms is shown schematically in **Figure 1**.

#### 2.1.1 Alloimmune response

The alloimmune response is the primary catalyst for the development of CAV. Graft endothelial cells, as the primary cells that interact with allogeneic antigens, are targeted by the recipient’s immune system shortly after transplantation (25). Antibody-mediated rejection (AMR) is a critical component of the alloimmune response that drives CAV progression. Jane-wit et al. demonstrated that panel reactive antibodies (PRAs) generated membrane attack complexes (MAC) on the surfaces of allogeneic vascular endothelial cells, increased the expression of inflammatory genes, and facilitated T-cell recruitment, ultimately advancing the development of CAV lesions (6). Simultaneously, B lymphocytes produce DSAs that bind to antigens on the surface of endothelial cells, forming immune complexes. These complexes activate the classical complement pathway via the C1 complex, leading to the formation of the MAC, which damages endothelial cells. This damage results in thrombosis and exposure of self-antigens, ultimately triggering an immune response against the graft (26). DSA characteristics, including persistent high titer, C1q-binding capacity, and specificity for human leukocyte antigen (HLA) class II antigens, are closely associated with an elevated risk of CAV (27–29). DSA deposition on endothelial cells triggers complement activation and elicits noncanonical NF-κB signaling. This process also recruits CD8^+^ cytotoxic T cells, indicating crosstalk between humoral and cellular alloimmunity (6, 30). In addition, DSA binding induces phenotypic changes in endothelial cells that promote vascular endothelial growth factor (VEGF) expression, smooth muscle proliferation, and neointimal formation (31–33). Clinical evidence indicates that late AMR is associated with a higher incidence of *de novo* coronary vasculopathy (34, 35).

Meanwhile, The canonical and noncanonical nuclear factor-κB (NF-κB) pathways have been implicated in CAV pathogenesis, coordinating the expression of proinflammatory cytokines, chemokines, and adhesion molecules that promote endothelial activation and leukocyte recruitment (36). Particularly, the noncanonical NF-κB signaling pathway is activated during this process, which is observable in human allografts and xenografts (6). These observations indicate that blocking NF-κB signaling could aid in preventing disease progression. The donor organ and its resident cells also actively shape the alloimmune response that drives CAV rather than serving solely as passive targets. Donor vascular endothelial cells could present alloantigens to recipient CD4^+^ T cells and, through a programmed death ligand 1 (PD-L1)-dependent pathway, induce Treg differentiation that modulates the local immune environment (37). Whether this donor-recipient immune crosstalk is altered during CAV progression remains to be further investigated.

Besides, Cai et al. employed single-cell RNA sequencing (scRNA-seq) to identify 21 cell clusters throughout several disease phases of transplant arteriosclerosis in a mouse model of CAV and reported that local tertiary lymphoid structures and the allogeneic immune response were pivotal in CAV pathology (38). Lymphatic endothelial cell chemotaxis and immune cell infiltration are regulated by the C-C motif chemokine ligand 21 (CCL21) and its C-X-C motif chemokine receptor 3 (CXCR3). *In vivo* investigations have demonstrated the therapeutic potential of CCL21 and CXCR3-neutralizing antibodies (38). These results underscore the pivotal role of alloimmune responses in the onset and progression of CAV.

#### 2.1.2 Inflammatory microenvironment

Inflammation is a critical factor in the development of CAV. Transplant damage, ischemia–reperfusion injury and sustained allogeneic immune responses could contribute to the establishment of a localized inflammatory milieu within the transplant (7). Activated endothelial cells upregulate adhesion molecules, including vascular cell adhesion molecule-1 (VCAM-1) and intercellular adhesion molecule-1 (ICAM-1), and secrete chemokines, such as CCL2 and CCL5, that recruit leukocytes to infiltrate the graft and intensify the inflammatory response (7, 39–41). Macrophages are the primary immune cells that infiltrate the graft and exhibit significant plasticity in their phenotype and function (42). Chen et al. reported that macrophage-specific knockout of the *Mek1/2* gene could safeguard the graft from rejection, reduce the proinflammatory and glycolytic capabilities of allograft-infiltrating macrophages (AIMs), and downregulate PKM2 expression during cardiac transplantation rejection, as demonstrated through scRNA-seq and flow cytometry (43). These findings indicate that the MEK1/2-PKM2 pathway serves as a crucial regulatory node for macrophage immunometabolic reprogramming and holds potential therapeutic significance. DSA-mediated CAV requires the active engagement and effector functions of recipient NK cells, while the mere presence of DSAs is insufficient to induce vascular pathology (44). NK cells could engage DSAs via the Fc receptor CD16a, thereby triggering chronic rejection and antibody-dependent cytotoxicity (45–47). After heart transplantation, NK cells are activated upon recognition of major histocompatibility complex class I (MHC-I) expressed on the allograft, leading to the secretion of effector molecules such as TNF-α and IFN-γ (48). Notably, IFN-γ not only participates in T lymphocyte-mediated acute rejection but also further upregulates the expression of multiple inflammatory factors, thereby exacerbating endothelial inflammatory injury (49). Furthermore, proinflammatory cytokines, including IL-6, show a significant association with the progression of CAV (50–52). Muckenhuber et al. demonstrated that the simultaneous administration of anti-thymocyte globulin (ATG) and IL-6-targeted inhibition via cytotoxic T lymphocyte-associated protein 4-immunoglobulin fusion protein (CTLA-4-Ig) treatment could successfully surmount costimulation blockade-resistant rejection by increasing local immune control of the graft. The effectiveness of this technique was experimentally validated to rely on the presence of Tregs, as their removal resulted in the loss of the protective effect (53). These works offer critical evidence for clarifying the inflammatory signal-induced CAV.

#### 2.1.3 Phenotypic switching of VSMCs

The phenotypic switching and aberrant activation of VSMCs are critical pathological hallmarks throughout CAV, with their activation, migration, and proliferation representing the cytological foundation of CAV (54). Driven by inflammation and the immune response, VSMCs undergo phenotypic transformation, dedifferentiating from a contractile to a synthetic phenotype (55, 56), migrating from the media to the intima, and abnormally proliferating and producing a large amount of extracellular matrix, eventually leading to diffuse thickening of the intima and the formation of typical CAV lesions (7, 57). In this process, endothelial injury and dysfunction play initiating roles: damaged endothelial cells not only directly participate in local inflammatory reactions but also release mediators such as platelet-derived growth factor (PDGF) (58–61), promote and accelerate the proliferation and migration of VSMCs. Among these mediators, early growth response factor-1 (Egr-1) responds to alloantigen stimulation to regulate the expression of target genes such as PDGF-A and transforming growth factor-β (TGF-β), thereby promoting intimal hyperplasia (62). Thrombospondin-1 (TSP-1) drives the characteristic vascular remodeling of CAV by inhibiting angiogenesis and promoting VSMC proliferation (63). In addition, expression of allograft inflammatory factor-1 (AIF-1) is positively correlated with both the severity of rejection and VSMC proliferation (64). Persistent post-transplant immune inflammation is a crucial mechanism driving the phenotypic switching of VSMCs. The recruitment of inflammatory cells, such as neutrophils, mediated by innate immunity, induces VSMC injury, and inhibition of this process could reverse their pathological activation (65–67). Furthermore, cytomegalovirus (CMV) infection directly upregulates MHC-I on the surface of VSMCs, thereby enhancing their immunogenicity (68). Similarly, autoimmune responses could promote VSMC proliferation and intimal hyperplasia (69). Both mechanisms contribute to the pathological progression of VSMC phenotypic modulation.

Phenotypic switching of VSMCs in CAV is regulated at multiple levels. This multilayered regulation includes the antagonistic and nonredundant functions of the histone acetyltransferases p300 and CREB-binding protein (CBP) (70), as well as inhibition of apoptosis mediated by erythroblast transformation-specific 2 (Ets-2) (71). In addition, aberrant activation of signaling axes involving AXL receptor tyrosine kinase (72), matrix metalloproteinase-14 (MMP-14)/CD44 (73), and cytochrome p450 (CYP) 2C (74) further contributes to this process. These mechanisms collectively determine the pathological phenotype of VSMCs.

#### 2.1.4 Shared pathological features of chronic vascular rejection across other allografts

Chronic vascular rejection in solid organ and vascularized composite allografts shares several pathological features with CAV, including endothelial injury, vascular remodeling, and immune dysregulation. A common morphological characteristic is diffuse intimal hyperplasia with secondary luminal narrowing, which has been consistently documented in heart, kidney, liver, intestinal, and vascularized composite allografts (21, 75, 76). VSMC migration from the media into the intima, accompanied by phenotypic switching, represents the cellular basis of this intimal thickening (77). Endothelial cells serve as early targets of alloimmunity in both heart and kidney transplant models, where they acquire a proliferative and proinflammatory phenotype through mammalian target of rapamycin (mTOR)/Akt signaling (78, 79). In kidney transplantation, NK cells mediate endothelial injury through missing-self recognition in an mTORC1-dependent manner (80). Meanwhile, Humoral immunity represents a common mechanism driving chronic rejection across organ types, with DSA-induced complement activation and C4d deposition serving as reproducible markers of chronic rejection in heart, kidney, and liver allografts. Antibody-mediated endothelial injury has also been documented during long-term follow-up of vascularized composite allografts (81–83). Therapeutic strategies targeting endothelial stability, smooth muscle proliferation, and Treg-mediated immune regulation may offer cross-organ benefits, although their clinical efficacy requires further investigation (84, 85).

### 2.2 Progression in the diagnosis of CAV

Early detection of CAV is essential for improving prognosis and timely intervention. At present, coronary angiography is regarded as the gold standard for diagnosis; nonetheless, this technique is limited to displaying the extent of arterial lumen stenosis and exhibits reduced sensitivity to early diffuse lesions (86). Intravascular ultrasound (IVUS) offers greater insights into artery wall structure and is regarded as one of the most sensitive techniques for identifying CAV. However, its invasiveness restricts its regular clinical use (87).

#### 2.2.1 Noninvasive imaging diagnosis

The ongoing advancement of imaging technology and biomarker research has led to the steady emergence of many noninvasive diagnostic approaches. Clerkin et al. demonstrated that among 181 heart transplant recipients who underwent PET assessment for CAV, the application of ^13^N-ammonia PET to evaluate the myocardial blood flow reserve (MBFR) had substantial prognostic significance for CAV, with a markedly elevated risk of mortality or retransplantation in patients with an MBFR ≤2.0 (2), indicating that microvascular CAV is independently linked to poor prognosis. A retrospective analysis by Albulushi et al. revealed that the global longitudinal strain (GLS) value progressively increased following heart transplantation, demonstrating high sensitivity in detecting early subtle myocardial alterations and indicating its potential utility to monitor posttransplant complications and CAV (88).

#### 2.2.2 Biomarkers

Olsen et al. reported that increased levels of donor-derived cell-free DNA (dd-cfDNA) correlated with the presence of DSAs and were likely linked to a greater incidence of CAV in individuals without acute rejection (AR) (89). Monitoring the variability of dd-cfDNA could reduce the false negative rate and increase its clinical utility in CAV and DSA monitoring. Rosello-Lleti et al. screened 228 fibrosis-related serum mRNAs and reported that 38 were differentially expressed in patients with moderate/severe (grade ≥2R) rejection, of which 16 molecules, such as RELB, TNS1, COL4A2 and JAK1, exhibited good discrimination ability in the diagnosis of moderate to severe rejection (90), suggesting that the serum mRNA levels of these genes are potential biomarkers for cardiac transplant rejection and CAV. Köster et al. demonstrated that high-sensitivity cardiac troponin and N-terminal pro-B-type natriuretic peptide (NT-proBNP) are significantly associated with CAV severity (91), highlighting their potential as noninvasive biomarkers for CAV surveillance. Almufleh et al. applied targeted aptamer proteomics to identify novel protein biomarkers for CAV (92), revealing multiple candidate proteins associated with CAV development. O’Hara et al. comprehensively summarized the current landscape of CAV biomarkers, emphasizing that emerging molecular markers may improve early detection and risk stratification for transplant recipients (93). These biomarkers encompass brain natriuretic peptide (BNP), circulating nucleic acids and angiogenesis-related mediators, such as VEGF-C, VEGF-A, and platelet factor (PF)-4.

#### 2.2.3 Computational pathology and gene expression profiling

Moreover, computational pathology and gene expression profiling offer novel methodologies for the prediction and diagnosis of CAV. The area under the receiver operating curve (AUROC) for the integrated iCAV-Pr model, developed by Peyster et al., was 0.93, highlighting the markedly increased predictive ability for CAV (94). Abdrakhimov et al. identified 809 differentially expressed genes through gene expression profile analysis and isolated key gene markers such as HCP5, KLRD1, GZMB, PLA1A, GNLY, and KLRB1. The authors subsequently developed a machine learning model to predict acute cardiac rejection, which demonstrated high discrimination accuracy (95). Farcas et al. highlighted the significant negative predictive value of gene expression patterns in the diagnosis of heart transplant rejection and reported that this is anticipated to reduce the reliance on endomyocardial biopsy (EMB) (96).

While the assessment and diagnosis of cardiac allograft rejection, including CAV, continue to depend on EMB histological evaluation, the emergence of noninvasive techniques is progressively transforming the monitoring approach (97). **Table 1** summarizes the progression in the diagnostic modalities for CAV, highlighting the shift from invasive to non-invasive techniques.

**Table 1:** Progression in the diagnosis of CAV.

Diagnostic method	Core principle/technology	Invasive/non-invasive	Advantages	Disadvantages/challenges	References
Coronary angiography	Vascular cavity morphology assessment	Invasive	Gold standard; intuitive	Low sensitivity to early diffuse lesions; unable to assess vascular wall pathology	(6)
Intravascular ultrasound (IVUS)	Vessel wall structure imaging	Invasive	Most sensitive method for assessing vessel wall; used to ascertain the extent of intimal growth	Complex operation; high cost	(7)
^13^N-ammonia PET	Myocardial blood flow reserve (MBFR) assessment	Non-invasive	Evaluate microvascular CAV; significant prognostic value	Radiation exposure; costly apparatus; limited adoption	(2)
Global Longitudinal Strain (GLS)	Echocardiographic myocardial deformation analysis	Non-invasive	High sensitivity; capable of identifying early myocardial changes	Require a standardized benchmark; operator-dependent	(88)
Donor-derived cell-free DNA (dd-cfDNA)	Circulating DNA fragment detection	Non-invasive	Serve as an indicator of graft rejection and injury	False negative rate; the specificity requires enhancement; additional indicators must be incorporated	(89)
Serum fibrosis-related mRNA	Gene expression profile analysis	Non-invasive	Identify molecular biomarkers of rejection	Currently in the research phase; extensive clinical validation is required	(90)
Computational pathology	Machine learning combined with histological analysis	Non-invasive	Enhance predictive and diagnostic accuracy while reducing the reliance on biopsies	Large amounts of data are needed to train the model; explanatory challenges	(94, 95)

## 3 Role of Tregs in cardiac transplantation immunity

**Figure 2:**
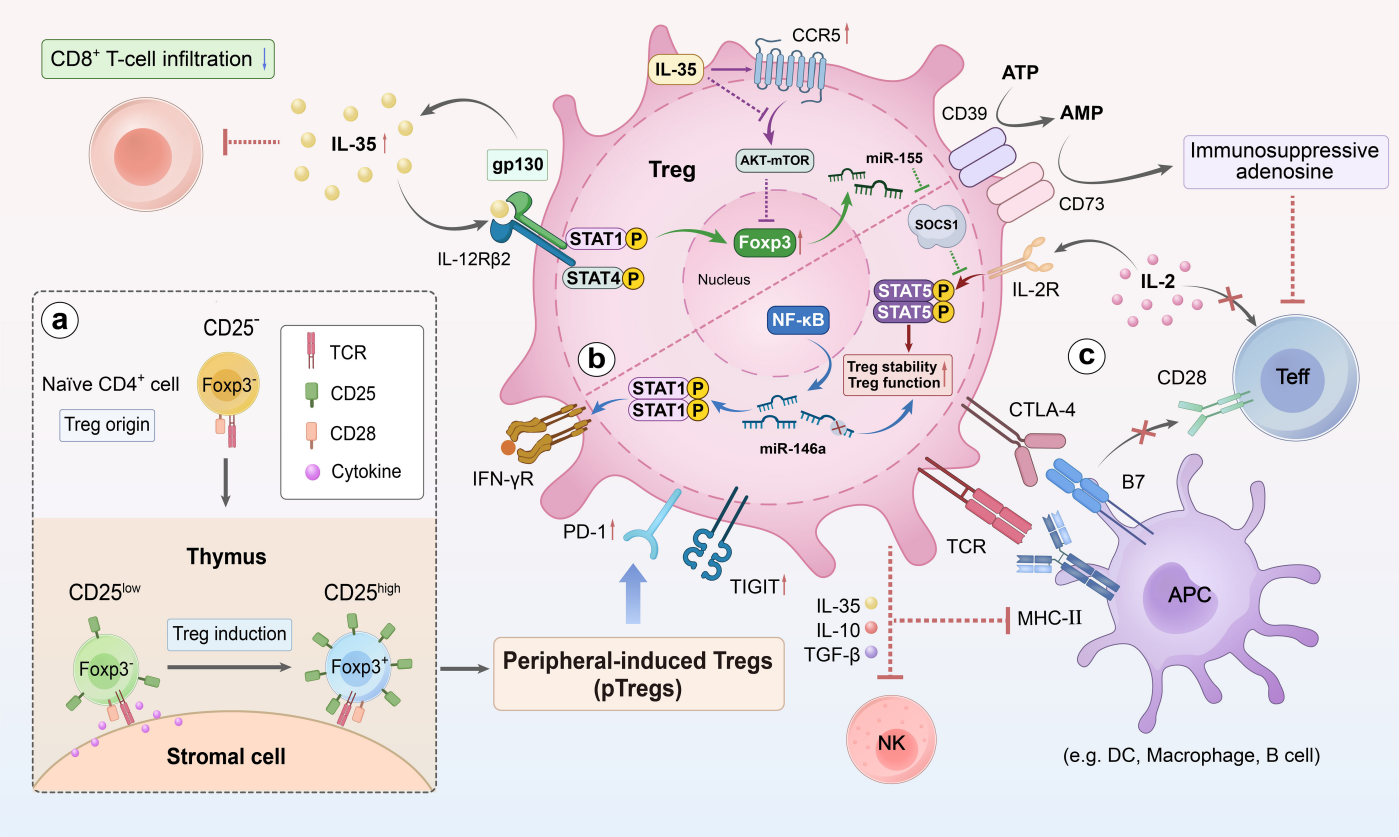
Treg phenotype, heterogeneity, and immunosuppressive mechanisms in cardiac transplantation: **(a)** Treg origin and induction. Naïve CD4^+^ T cells (CD25^-^Foxp3^-^) differentiate into thymus-derived natural Tregs (tTregs) through high-affinity TCR recognition of MHC-self-peptide complexes presented by stromal cells, acquiring Foxp3 expression (Foxp3^+^) and elevated CD25 levels (CD25^high^), and alternatively differentiate into peripheral-induced adaptive Tregs (pTregs) in peripheral lymphoid organs. **(b)** Factors maintaining the Treg phenotype. Cytokine and microRNA cooperatively stabilize Treg phenotype. IL-2 activates the STAT5 pathway to sustain Foxp3 expression, while IL-35 binds gp130 to activate STAT1, creating a positive feedback loop that enhances both Foxp3 stability and IL-35 secretion. Additionally, IL-35 facilitates CCR5-mediated migration, suppresses the Treg-weakening AKT-mTOR pathway, and upregulates immune checkpoints including PD-1 and TIGIT. At the post-transcriptional level, miR-155 promotes functional integrity by inhibiting SOCS1, a negative regulator of STAT5. miR-146a regulates Treg function through the IFN-γ/STAT1 pathway. Ablation of miR-146a promotes Treg proliferation and prolongs graft survival but weakens Th1 suppression. **(c)** Immunosuppressive mechanisms of Tregs. Tregs utilize multiple ways to maintain immune tolerance and mitigate graft rejection. Through the competitive sequestration of IL-2 via high-affinity receptors and the CD39/CD73-mediated hydrolysis of extracellular ATP into adenosine, Tregs induce metabolic disruptions that deprive Teffs of essential activation signals. Simultaneously, Treg-expressed CTLA-4 blocks B7 molecules on APCs, preventing CD28-mediated costimulation and limiting Teff priming. This inhibitory effect is further supported by the downregulation of MHC-II and costimulatory molecules on APCs, which reduces their antigen-presentation capacity. These actions collectively suppress Teff and NK cell functions, restrict CD8^+^ T-cell infiltration, and prolong cardiac allograft survival.

Tregs are considered promising therapeutic targets for fostering graft tolerance and preventing acute and chronic rejection after cardiac transplantation (25, 98). **Figure 2** shows Treg phenotype, heterogeneity, and its immunosuppressive mechanisms in CAV.

### 3.1 Treg phenotype and heterogeneity

Tregs are a subpopulation of CD4^+^ T lymphocytes distinguished by the expression of the transcription factor Foxp3. As the principal transcription factor, Foxp3 is crucial for the formation, functional differentiation, and lineage stability of Tregs (99, 100). Tregs characteristically express elevated levels of CD25 (IL-2 receptor α chain) and CTLA-4 but exhibit reduced levels of CD127 (IL-7 receptor α chain) in conjunction with Foxp3 (101–103). The decreased expression of CD127 serves as a critical marker for differentiating Tregs from activated Teffs, as CD127 expression is negatively correlated with both Foxp3 expression and the inhibitory function of Tregs (101). Tregs are primarily classified into two categories: thymus-derived natural Tregs (tTregs or nTregs) and peripheral-induced adaptive Tregs (pTregs or iTregs) (104–106). tTregs originate in the thymus and perform their inhibitory function by detecting MHC-self-peptide complexes with high affinity, which underpins the maintenance of self-tolerance (106, 107). pTregs are derived from conventional CD4^+^ T cells in peripheral lymphoid organs under the influence of specific microenvironments (e.g., TGF-β, IL-2, and retinoic acid) and function primarily at inflammatory sites to regulate local immune responses (104, 108). Yadav et al. reported that pTregs and tTregs exhibit functional and T-cell receptor (TCR) repertoire differences and could be differentiated by distinct cell surface markers and transcription factors, such as Neuropilin-1 (Nrp-1) and Helios (105), suggesting that pTregs play a specific role in the inflammatory milieu. In recent years, the advent of scRNA-seq technology has significantly enhanced researchers’ understanding of Treg heterogeneity. Chen et al. employed scRNA-seq to systematically examine CD45^+^ immune cells in mouse heart allografts and elucidated the dynamic alterations in immune cells and the local intercellular communication network that occur during transplant rejection while identifying critical alloreactive cell subsets, including activated Tregs (109).

### 3.2 Factors maintaining the Treg phenotype

Tregs maintain immune homeostasis by suppressing the activation and proliferation of Teffs. However, under inflammatory conditions or specific stimulatory cues, a subset of Foxp3^+^ Tregs may lose Foxp3 expression and differentiate into ex-Tregs that acquire an Teff-like phenotype and function (110). These ex-Tregs not only exhibit diminished immunosuppressive capacity but also mediate aberrant immune responses, thereby contributing to various pathological processes. Collectively, these observations underscore the marked phenotypic plasticity of Tregs.

#### 3.2.1 Cytokine

Cytokine-mediated signal transduction represents a core pathway in maintaining the phenotypic stability of Tregs. Among these signals, IL-2 serves as a crucial modulator by activating the signal transducer and activator of transcription 5 (STAT5) pathway, which sustains continuous Foxp3 expression and regulates Treg survival and proliferation (111, 112). Disruption of IL-2 signaling directly leads to alterations in the Treg phenotype (113). TGF-β, on the other hand, induces the differentiation of peripheral naïve CD4^+^ T cells into iTregs (114). Accordingly, blockade of TGF-β signaling or overexpression of its negative regulator SKI compromises Treg stability. Notably, ARKADIA, an E3 ubiquitin ligase targeting SKI, effectively rescues this defect by promoting SKI degradation (115). Within the microenvironment, M2 macrophages enhance Treg phenotypic stability and suppressive function through paracrine TGF-β signaling, which upregulates Foxp3 expression in iTregs (116). IL-2 and TGF-β signaling act synergistically to regulate Treg differentiation and phenotypic maintenance (117). For instance, DNA methyltransferase inhibitor together with endogenous TGF-β/IL-2 signaling promotes the stable expression of Foxp3 in peripheral CD4^+^Foxp3^-^ T cells, leading to their differentiation into fully functional iTregs (118). Meanwhile, IL-27 enhances the immunosuppressive function of Tregs, augments their capacity to negatively regulate inflammatory Teffs. Conversely, deficiency in the IL-27 receptor α chain (IL-27Rα) results in impaired Treg function (119, 120). IL-6, by contrast, exerts opposing effects by inhibiting Treg proliferation and downregulating Foxp3 expression, thereby increasing the risk of phenotypic conversion of Tregs within inflammatory microenvironments (121).

#### 3.2.2 microRNAs

MicroRNAs (miRNAs) serve as fine-tuned regulators by orchestrating Treg differentiation, phenotypic maintenance, and immunosuppressive function through multi-target modulation, particularly within the contexts of autoimmune responses and inflammatory microenvironments (122). For example, miR-155 promotes Treg development and functional integrity by inhibiting the expression of suppressor of cytokine signaling 1 (SOCS1) (123). In addition, miR-10a or miR-182 sustains immune homeostasis by regulating the differentiation of Th1- or Th2-associated Tregs (124). miR-29a-3p contributes to the regulation of the Th17/Treg differentiation and preserves the immunosuppressive capacity of Tregs within corresponding inflammatory milieus (125).

Cytokine signaling and miRNAs crosstalk to maintain the Treg phenotype. miR-125a-5p inhibits IL-6 signaling, which reduces Treg sensitivity to pro-inflammatory cytokines (126). TGF-β-induced Tregs release miR-449a via extracellular vesicles to target Notch1, further enhancing the phenotypic stability (127). Meanwhile, TGF-β upregulates miR-10a, blocking the differentiation of iTregs into follicular helper T cell (Tfh)-like cells (128).

### 3.3 Immunosuppressive mechanism of Tregs

Tregs are the principal regulators of immunological homeostasis and the induction of immune tolerance. Tregs efficiently suppress the activation, proliferation, and effector function of Teffs through synergistic effects across multiple pathways (16, 17). The precise mechanisms include the release of inhibitory cytokines, cytotoxic effects, metabolic disruption, expression of inhibitory receptors, and direct modulation of antigen-presenting cell (APC) function. At the cytokine level, IL-10 secreted by Tregs could significantly inhibit the proliferation of and cytokine production by Teffs and negatively regulate the function of APCs (129). Additionally, TGF-β not only exerts pivotal modulatory effects on the biological balance between Teffs and Th17 cells (130), but studies have also shown that the CD4^+^CD25^+^ Treg subset could directly mediate immunosuppression in a cell contact-dependent manner with the help of membrane-bound TGF-β1 (131, 132). Tregs competitively consume IL-2 in the microenvironment through high-affinity IL-2 receptors and hydrolyze extracellular ATP into adenosine, which has immunosuppressive activity, with the assistance of CD39/CD73 ectoenzymes expressed on their surface. These functions deprive Teffs of the metabolic signals required for their activation (16). CTLA-4, which is abundantly expressed by Tregs, could competitively bind to B7 molecules on the surface of APCs alongside CD28, obstructing the costimulatory signaling pathway and thereby limiting the initial activation of Teffs (133). Furthermore, Tregs could directly influence APCs by downregulating the expression of MHC-II and costimulatory molecules, thus decreasing their antigen presentation capacity and indirectly suppressing the immunological response of Teffs (134, 135).

### 3.4 Stability and functional regulation of Tregs

The stability and function of Tregs are crucial for their immunosuppressive function. Liu et al. reported that the absence of Mettl14 reduces N6-methyladenosine (m6A) modification levels in Tregs, which adversely affects FoxP3 expression and compromises Treg functionality, consequently worsening transplant rejection (136). Lu et al. demonstrated that the ablation of miR-146a in a murine heart transplantation model promotes Treg proliferation and *in vitro* immunosuppression, which extends graft survival. However, these effects also weaken the suppression of Th1 immune responses. Mechanistic studies have indicated that miR-146a regulates Treg function via the IFN-γ/STAT1 pathway. Co-inhibition of IFN-γ/STAT1 signaling improves transplant outcomes and alleviates the dysfunction of Tregs associated with miR-146a deficiency (137). These studies elucidated the intricate molecular mechanisms regulating Treg function and stability and presented novel targets for the advancement of Treg-focused therapies.

### 3.5 Tissue-resident Tregs in the transplant microenvironment

Tissue-resident Tregs are crucial regulators of the local graft immune microenvironment that influence CAV-related vascular inflammation. Compared with circulating Tregs, they are defined by long-term survival, tissue adaptation, and local effector function within nonlymphoid organs (138). Their resident phenotype is shaped by coordinated signaling and metabolic programs. Ca^2+^ release-activated Ca^2+^ (CRAC) channel-mediated calcium signaling contributes to the differentiation of mature Treg into tissue-resident subsets (139), and mitochondrial transcription factor A (Tfam)-dependent oxidative phosphorylation supports their persistence in vascular and other nonlymphoid tissues (140). Tumor necrosis factor receptor 2 (TNFR2) costimulation further promotes the conversion of tTregs into tissue-resident effector Tregs (141). Treg residency is also tissue specific. In cardiovascular tissues, resident Tregs suppress Teff proliferation, reduce Th17 infiltration, and help maintain vascular wall homeostasis (142). In transplantation, infused donor-derived Tregs rapidly acquire tissue-residency gene programs matching the recipient organ, and donor-specific transfusion combined with CD40L blockade promotes the progressive and sustained accumulation of Tregs in lymphoid organs and hepatic tissue (143, 144). These findings indicate that Tregs act not only through systemic immunosuppression but also through local tissue adaptation (145), offering a potential route to stabilize graft vascular immunity.

### 3.6 Targeting Tregs to mitigate rejection and prolong the viability of cardiac transplants

Tregs exhibit considerable promise in heart transplantation by facilitating immunological tolerance to allogeneic grafts, hence reducing the rates of acute and chronic rejection (146). Pilat et al. demonstrated that the infusion of Tregs into a mouse model of mixed chimerism effectively inhibited chronic rejection of allogeneic heart grafts, markedly improving the efficacy and safety of the transplantation protocol (19). Schwarz et al. further confirmed that *in vivo* Treg expansion mediated by IL2/anti-IL2 complexes partially reversed graft damage during CTLA-4-Ig treatment, significantly prolonging allogeneic graft survival and improving histopathological outcomes (20). Ravichandran et al. revealed that intervention with low-dose IL-2 (2000 IU/day) beginning on day 14 after transplantation markedly extended the survival of chronic allogeneic cardiac grafts in mice (>100 days). This phenomenon was associated with the increased infiltration of CD4^+^CD25^+^Foxp3^+^ Tregs in the spleen and graft site and circulating levels of Foxp3^+^ exosomes, indicating that IL-2 mitigates CAV or chronic rejection by inducing Tregs and exosomes (147).

Furthermore, IL-35 enhances the immunosuppressive function of splenic Tregs in *ApoE^-/-^* mice and promotes their migration into graft vessels by inducing the expression of anti-inflammatory C-C chemokine receptor type 5 (CCR5) on Tregs, facilitating CCR5-mediated Treg migration, suppressing the Treg-weakening AKT-mTOR pathway, and upregulating the Treg-supporting immune checkpoint receptors programmed death-1 (PD-1) and T cell Ig and ITIM domain (TIGIT) (148). Huang et al. also demonstrated that IL-35 increases the Treg phenotype and functional stability, as well as its IL-35 secretion, by decreasing CD8^+^ T-cell infiltration and increasing the proportion and expression of Foxp3^+^ Tregs, therefore generating a positive feedback loop that extend cardiac transplant longevity. These effects are intricately linked to the activation of the gp130/STAT1 signaling pathway by IL-35 in Tregs (149).

## 4 Disruption of Treg homeostasis in the CAV microenvironment

Tregs maintain immune homeostasis through multilayered suppressive mechanisms that include inhibitory cytokine secretion, metabolic competition with Teffs, and direct modulation of APC function (150). Under homeostatic conditions, IL-2/STAT5 and TGF-β/Smad signaling cooperatively maintain Foxp3 expression and suppressive capacity (111, 112, 114). However, the CAV microenvironment is fundamentally distinct. Persistent alloantigen exposure, sustained endothelial activation, and progressive metabolic stress collectively generate a milieu in which the suppressive machinery that defines Treg identity is systematically undermined (25, 151, 152). Tregs are incapacitated, transforming cells that should enforce graft tolerance into dysfunctional bystanders.

### 4.1 Foxp3 instability and metabolic compromise of Tregs

A defining characteristic of Treg dysfunction in CAV is the instability of Foxp3 (110), which destabilizes the Treg lineage, impairs their capacity to suppress Teff responses (152, 153). Roldan et al. established that Treg biology and Foxp3 are dysregulated in heart transplant recipients who develop CAV, demonstrating that the equilibrium between Tregs and effector cells is shifted in the context of transplant vasculopathy (154). Mengrelis et al. showed evidence that Foxp3^+^ Tregs exhibit phenotypic instability under the inflammatory conditions of the cardiac allograft (151). Wang et al. demonstrated that Treg function declines continuously throughout the post-transplant phases, marked by progressive Foxp3 downregulation (153). Tregs are also metabolically compromised in CAV. Physiologically, Tregs rely on fatty acid oxidation (FAO) and mitochondrial oxidative phosphorylation (OXPHOS) for energy, whereas Teffs depend on glycolysis (155, 156). Within the inflamed allograft, Teffs compete with Tregs for limited nutrients while hypoxic conditions compromise OXPHOS, a pathway upon which Tregs rely for suppressive function (157, 158). Mendes et al. demonstrated that oral tributyrin, a short-chain fatty acid (SCFA) prodrug that supports FAO, enhances Treg-mediated immune regulation and prolongs allograft survival in preclinical models (159).

### 4.2 Impaired Treg trafficking to the allograft and checkpoint dysregulation

In addition to intrinsic dysfunction, Tregs in CAV face barriers to graft access and persistence (151, 152). Yang et al. identified impaired *in vivo* delivery and persistence as primary obstacles to Treg efficacy in solid organ transplantation (152). Saxena et al. demonstrated that Treg interactions with lymphatic endothelial cells are essential for tissue stability and allograft survival (160). In CAV, endothelial injury and disruption of lymphoid structures within the allograft perturb Treg distribution, producing a local Treg-to-Teff imbalance (151, 160, 161). Moreover, the checkpoint-dependent suppressive machinery of Tregs undergoes dysregulation in the alloreactive milieu of CAV. The centrality of checkpoint molecules to CAV pathogenesis is underscored by clinical evidence that pharmacologic PD-1 blockade with pembrolizumab triggers fatal CAV and confirms that intact coinhibitory signaling is essential for preventing transplant vasculopathy (162). Grant et al. systematically examined the checkpoint inhibitor treatment in solid organ transplant recipients and concluded that PD-1 inhibition is associated with a higher risk of allograft rejection than CTLA-4 inhibition, corroborating the essential role of the PD-1 pathway in maintaining graft immune homeostasis (163). Whether and how the expression of checkpoint molecules on intragraft Tregs is altered in CAV warrants further investigation. Elucidating the dynamic changes in the Treg checkpoint landscape during chronic rejection represents a promising direction for understanding the immune mechanisms that drive transplant vasculopathy and for identifying new therapeutic targets.

### 4.3 Immunosuppressant-mediated disruption of Treg homeostasis

Immunosuppressive agents used for CAV prevention and treatment also disrupt endogenous Treg homeostasis. Calcineurin inhibitors (CNIs) and mTOR inhibitors appear to have divergent effects on Treg biology. Overall, mTOR inhibition is more favorable for Treg maintenance. Rapamycin suppresses glycolysis in Th17 cells while promoting FAO in Treg, thereby modulating the Th17/Treg balance (164). In nonhuman primate models, rapamycin preserved Foxp3 and CD25 expression in Treg, whereas tacrolimus failed to maintain Foxp3 expression (165). Clinical studies also showed increased peripheral Treg numbers after conversion from CNI-based regimens to mTOR inhibitors in liver and kidney transplant recipients (166, 167). By contrast, prolonged CNI exposure reduces Treg suppressive function while CNI withdrawal helps preserve Treg frequency (168–170). These observations support Treg-guided adjustment of immunosuppression and suggest that mTOR inhibitors may serve as favorable partners for Treg-based therapy.

## 5 Regulatory mechanisms of Treg-associated signaling pathways in CAV

**Figure 3:**
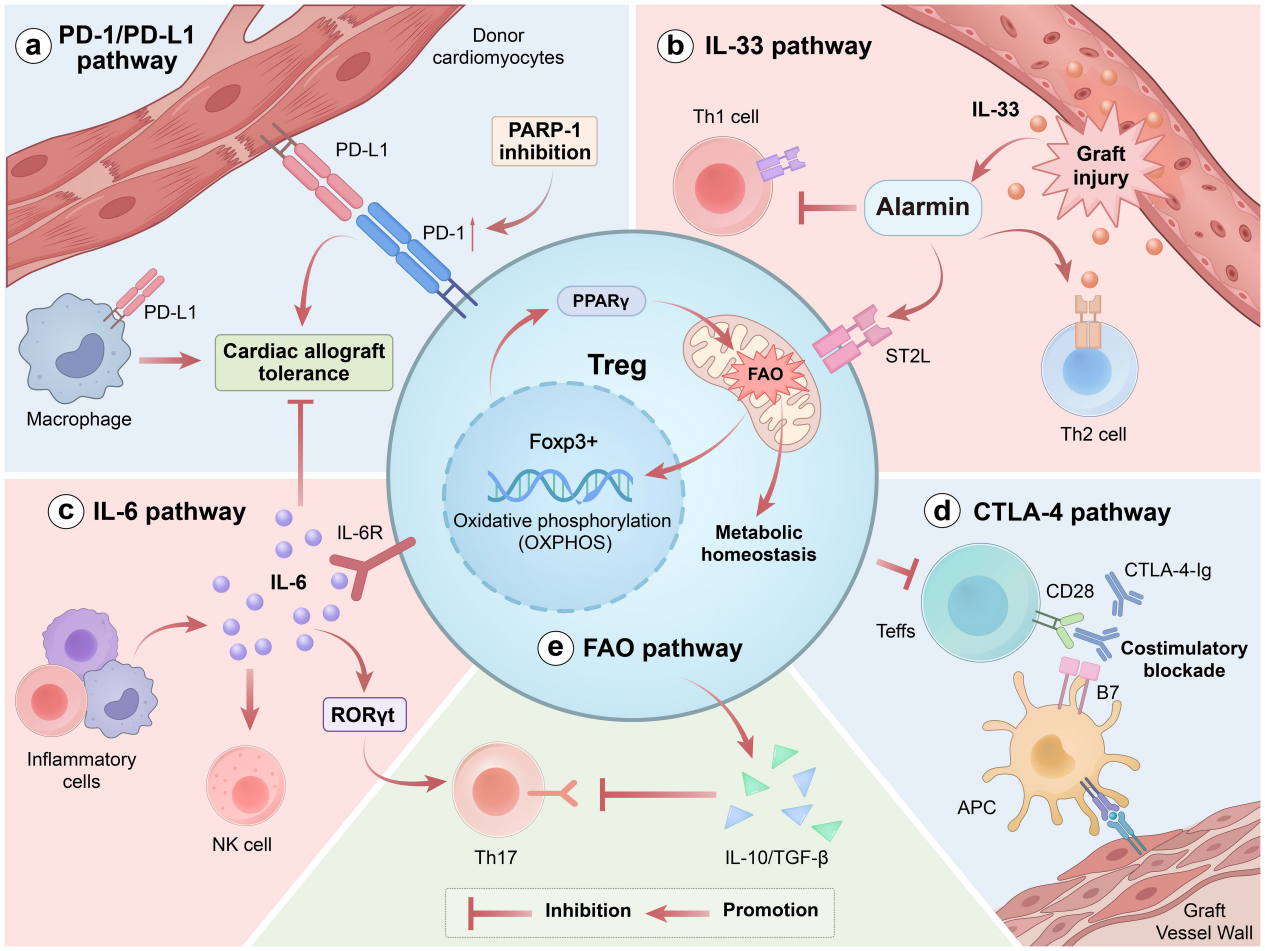
Regulatory mechanism of Treg-associated signaling pathways in CAV: **(a)** PD-1/PD-L1 pathway. Donor-derived PD-L1, expressed on cardiomyocytes and M2 macrophages, engages PD-1 on Tregs to sustain peripheral immune tolerance and restrict Teff activation. PARP-1 inhibition augments PD-1 expression, thereby improving Treg stability and promoting cardiac allograft tolerance. **(b)** IL-33 pathway. Released passively as an alarmin after graft injury, IL-33 binds the ST2L receptor to increase Treg proliferation and immunosuppressive efficacy. By shifting immune responses toward Th2-type responses while suppressing Th1-type pathways, IL-33 mitigates inflammatory damage and prolongs allograft survival. **(c)** IL-6 pathway. Dysregulation of IL-6 signaling directly undermines Treg inhibitory function and disturbs the equilibrium between Tregs and inflammatory responses. IL-6 drives Th17 differentiation via RORγt and activates NK cells, both of which exacerbate CAV by promoting vascular intimal hyperplasia and facilitate graft rejection. **(d)** CTLA-4 pathway. CTLA-4-Ig inhibits Teff activation by obstructing the B7:CD28 costimulatory signaling pathway, thereby creating a milieu conducive to Treg survival and function. **(e)** Fatty acid oxidation (FAO) serves as a fundamental cellular metabolic process that is crucial for Treg function and metabolic homeostasis. PPARγ activation facilitates FAO and oxidative phosphorylation (OXPHOS), thereby sustaining Foxp3 expression and amplifying the immunosuppressive capacity of Tregs.

Treg-associated signaling pathways are essential for the onset and progression of CAV. These mechanisms preserve immunological homeostasis, mitigate excessive inflammatory responses, and suppress chronic rejection reactions such as CAV by regulating the recruitment, activity, and stability of Tregs (100). The regulatory mechanisms of these pathways in CAV are shown in **Figure 3**.

### 5.1 PD-1/PD-L1 pathway

The immunological coinhibitory pathway involving PD-1 and its ligand PD-L1 is crucial for sustaining peripheral immune tolerance, restricting Teff activation, and facilitating Treg homeostasis (171, 172). The PD-1/PD-L1 pathway regulates the equilibrium and self-limitation of the immune system by suppressing T-cell proliferation and the release of inflammatory factors, and is crucial for immune control and the development of CAV following heart transplantation (173). Research conducted by Yang et al. demonstrated that PD-L1 expression in the donor myocardium effectively suppresses the recipient T-cell response, reduces inflammatory cell infiltration, and postpones vascular intimal hyperplasia. Conversely, a lack of PD-L1 resulted in increased immune activation and significantly exacerbated CAV (174), indicating that donor-derived PD-L1 signaling is crucial for mitigating local inflammation and preserving vascular homeostasis. Tanaka et al. reported that PD-L1 deficiency decreases the number of Tregs and increases Teff activity, resulting in the breakdown of peripheral immunological tolerance and facilitating chronic rejection (175). Zhao et al. noted that mTOR-dependent M2 macrophages facilitate the preservation of Treg function through elevated PD-L1 expression, whereas PD-L1 inhibition disrupts this equilibrium, inducing a sustained inflammatory response and chronic rejection (176). Furthermore, in certain patients and murine models of long-term tolerance, total abrogation of established tolerance necessitates the concurrent elimination of Tregs, inhibition of PD-L1 expression, and infusion of a limited quantity of alloreactive T cells, thereby reinforcing the critical functions of these elements in sustaining immune homeostasis during cardiac transplantation (177).

Enhanced PD-1 signaling has the potential to substantially promote Treg proliferation, inhibit the inflammatory response, and delay the progression of CAV in numerous experimental models following heart transplantation. Dendritic cells exposed to the PD-L1-Ig fusion protein could facilitate the generation of Tregs and prolong the survival of cardiac allografts (178). Tregs that overexpress PD-1, in conjunction with CTLA-4-Ig, could markedly suppress Teff activation, proliferation, and inflammatory cytokine secretion by increasing the expression of inducible T-cell costimulator (ICOS) on Treg surfaces, which promote stable peripheral immune tolerance (179). Moreover, ATP-binding cassette member B5 (ABCB5)^+^ dermal immunoregulatory cells (DIRCs) could facilitate Treg production via the PD-1 pathway and prolong the survival of cardiac allografts (180). The poly(ADP-ribose) polymerase 1 (PARP-1) inhibitor AG14361 improves Treg stability by increasing PD-1 expression, consequently reducing cardiac transplant rejection (181). Targeting the PD-1/PD-L1 pathway is anticipated to emerge as a novel method for preventing CAV and increasing cardiac allograft tolerance.

### 5.2 IL-33 pathway

IL-33 is an important member of the IL-1 family. As a prototypical alarmin cytokine, IL-33 is secreted passively following tissue injury or necrosis (182, 183). Research has indicated that the IL-33/ST2 signaling pathway is crucial for sustaining immunological homeostasis and mitigating excessive inflammatory responses, particularly after cardiac allograft transplantation, when it plays an essential role in immunomodulation (184). IL-33 has been reported to increase the proliferation of CD4^+^Foxp3^+^ Tregs, thus facilitating immunological tolerance (185). Subsequent mechanistic investigations have demonstrated that the immunomodulatory role of IL-33 is contingent upon ST2L receptor-mediated signal transduction, which could increase the immunosuppressive efficacy of Tregs and facilitate their infiltration and persistence inside the graft. In a cardiac transplantation model, exogenous IL-33 administration markedly increased the proportion of Tregs in the graft and significantly prolonged graft survival (186), suggesting that IL-33 is pivotal for facilitating long-term transplantation success via a Treg-mediated immune tolerance mechanism.

IL-33 exerts an immunological protective effect in conjunction with Tregs by influencing the overall immune microenvironment. Research has indicated that IL-33 could enhance Th2-type immune responses while suppressing Th1-type immune pathways, therefore decreasing the secretion of proinflammatory molecules such as IFN-γ and mitigating inflammatory damage to cardiac allografts (187). In cardiac allografts lacking IL-33, arterial occlusion and fibrosis markedly intensified, whereas localized IL-33 injection substantially delayed the progression of CAV (188). The mechanism is thought to involve the modulation of macrophage polarization by IL-33, facilitating the transition of macrophages from the proinflammatory M1 phenotype to the reparative M2 phenotype (189). Moreover, IL-33 could augment the immunosuppressive capabilities of myeloid-derived suppressor cells (MDSCs), reduce inflammatory cell infiltration, and thereby preserve the local immunological homeostasis of the graft in conjunction with Tregs (187).

### 5.3 IL-6 pathway

Dysregulation of the IL-6 pathway directly undermines the inhibitory function of Tregs and disturbs the equilibrium between Tregs and inflammatory responses, which contributes to rejection and the progression of CAV (21, 53, 190). Muckenhuber et al. demonstrated in a murine heart transplant model that inhibiting IL-6 facilitates Treg accumulation in the graft, increases IL-10 expression, and markedly reduces local inflammation, thereby fostering long-term immunological tolerance (53). Similarly, Jagged2 signaling has been shown to reduce Treg function by increasing IL-6 levels, although the neutralization of IL-6 could counteract this effect and prevent rejection (191). Preliminary research has shown that anti-IL-6 monoclonal antibody therapy could prolong graft survival, reduce the levels of IFN-γ and IL-17, and increase the proportion of Tregs, indicating that the inhibition of IL-6 expression aids in reestablishing the immune equilibrium governed by Tregs (192). Additionally, Wu et al. demonstrated that digoxin antagonizes retinoic acid related orphan receptor γt (RORγt), a critical transcription factor that inhibits the conversion of Tregs to Th17 cells and preserves the state of immune tolerance, as it is downstream of the IL-6 signal that promotes Th17 differentiation (193).

During the chronic inflammatory phase of CAV, IL-6 originates from both systemic immune pathways and donor tissue. IL-6 produced from donors could increase Teff proliferation and impede apoptosis, worsening vascular inflammation and intimal hyperplasia, whereas the development of CAV is markedly reduced with donors lacking IL-6 (194). Moreover, inhibition of the IL-6/IL-6R signaling pathway could reduce the invasion of B cells, T cells, and macrophages, decrease complement activation levels, and improve the vascular architecture (195). Recent evidence indicates that increased plasma IL-6 levels correlate with intimal thickening and localized inflammatory responses in cardiac allografts, potentially serving as an inflammatory marker that is indicative of CAV (196). Moreover, the upregulation of IL-6 after ischemic injury could stimulate NK cells and intensify chronic vascular remodeling; furthermore, the transplantation of IL-6-deficient donor hearts into allogeneic *Rag^-/-^* (recombination activating gene, Rag) mice lacking T/B cells almost completely inhibited the occurrence of CAV (197). Suppressing the IL-6 signaling pathway or modulating its family members is anticipated to offer novel strategies for the prevention and treatment of CAV following heart transplantation through the reestablishment of Treg cell-mediated immunological tolerance.

### 5.4 CTLA-4 pathway

As an immunomodulatory protein, CTLA-4-Ig inhibits T-cell activation by obstructing the B7:CD28 costimulatory signaling pathway, consequently playing a crucial role in sustaining immunological homeostasis after heart transplantation and in the prevention and treatment of CAV (198, 199). The mechanism of action primarily involves decreasing Teff activation, blocking the production of proinflammatory mediators, and creating a milieu conducive to Treg survival and function (200). However, Riella et al. reported that inhibiting the B7:CD28 signaling pathway has been demonstrated to markedly decrease the quantity of Helios^+^ Tregs originating from the thymus (201). This effect has been associated with increased rejection risk in a cardiac transplantation model, which partially relies on Tregs. Notably, the immunomodulatory effect of CTLA-4-Ig is contingent upon dosage. Low-dose CTLA-4-Ig sustains tolerance primarily via a Treg-mediated active immunological modulation pathway. Upon the elimination of Tregs, the immunosuppressive efficacy of low-dose CTLA-4-Ig is compromised. High-dose CTLA-4-Ig monotherapy could sustain graft survival by directly suppressing Teff activity in a Treg-independent manner (202).

Recent investigations have shown the synergistic effects of CTLA-4-Ig in conjunction with other immune pathways. For instance, the administration of rapamycin following T-cell depletion has been reported to reduce the number of Tregs and facilitate the activation of Tfhs, resulting in antibody-mediated vascular damage. The concurrent use of CTLA-4-Ig could ameliorate this detrimental effect by suppressing Tfh activity and decreasing vascular inflammation in the graft (203). Moreover, inhibition of IL-6 could increase the immunotolerance effect of CTLA-4-Ig, markedly increase the proportion of Tregs and expression of IL-10 in the graft, mitigate the inflammatory response, and facilitate long-term rejection control (53). Similarly, the utilization of the IL-2 complex could counteract the reduction in Treg numbers induced by CTLA-4-Ig, markedly improving the vascular architecture and functionality of the graft (20). Furthermore, Erythropoietin (EPO) could stimulate anti-inflammatory signaling in myeloid cells, collaboratively increasing Treg proliferation alongside CTLA-4-Ig and further mitigating CAV-like vascular remodeling (204). However, in the context of metabolic disorders, such as hyperlipidemia, Treg dysfunction could lead to resistance to the immunotolerance effect of CTLA-4-Ig, thereby exacerbating vascular inflammation and promoting CAV progression (205).

### 5.5 FAO pathway

Fatty acid oxidation, which serves as a fundamental cellular metabolic process for energy production, is crucial for the preservation and modulation of Treg function (206, 207). FAO could sustain the metabolic homeostasis of Tregs and increase their immunosuppressive capacity, hence aiding in the preservation of immunological homeostasis and preventing excessive immune responses (208). Moreover, PPARγ activation could selectively increase the expression of CD36 and carnitine palmitoyltransferase 1 (CPT1), facilitate FAO, and increase the surface levels of TβRII and IL-2Rα on the Treg plasma membrane, thereby further amplifying the immunosuppressive capabilities of Tregs (209). These findings offer novel research avenues for modulating immune responses via metabolic pathways.

Research on the mechanisms of FAO and its associated metabolic pathways in cardiac transplantation immunity and CAV remains insufficient. Previous research indicated that FAO could promote immunological tolerance in chronic transplant immune responses by increasing OXPHOS in Tregs (210). Notably, the liver could control cardiac allograft rejection through the PCSK9/CD36 pathway (211), indicating that donor heart–liver crosstalk is crucial for immunological homeostasis remodeling following heart transplantation. However, whether FAO could modulate Treg function to mitigate CAV following cardiac transplantation is unclear. Therefore, modulating FAO represents a novel immunomodulatory approach for heart transplantation and serve as a potential therapeutic target for mitigating CAV.

## 6 Therapeutic potential of Treg-based therapy in CAV

Treg-directed therapy is intended to increase long-term graft survival and decrease reliance on conventional immunosuppressants through the expansion of Tregs or the augmentation of their functional capacity (212, 213). This strategy optimizes immune tolerance maintenance, reduces rejection reactions, and minimizes related complications after transplantation, thereby improving patient quality of life and long-term graft prognosis.

### 6.1 Autologous Treg therapy

Tregs are essential for sustaining peripheral immune tolerance and preventing transplant rejection (214). The pathogenesis of CAV is intricately associated with Treg depletion and dysfunction (215), and increased Treg homeostasis is regarded as crucial for improving long-term graft survival. Autologous Treg therapy involves the *in vitro* expansion of the recipient’s own Tregs, followed by their reinfusion to restore immune balance. This approach fundamentally regulates the inflammatory response rather than merely suppressing immune activity, positioning it as a potential strategy for the prevention and reversal of CAV (25). Tsang et al. was the first to confirm this concept. The combination of short-term immunosuppressants and CD4^+^CD25^+^ Tregs with indirect alloantigen specificity in a mouse heart transplantation model has shown that it is possible to achieve long-term tolerance of heart allografts and significantly slow vascular intimal hyperplasia. These findings suggest that Tregs could inhibit CAV-like inflammatory responses through antigen-specific pathways (216). Nevertheless, in nonhuman primate trials, *in vitro*-expanded darTregs (donor antigen-alloreactive Tregs) exhibited promising inhibitory activity prior to transplantation. However, essential molecules, including Foxp3 and CTLA-4, were rapidly lost after transplantation, leading to immune regulation failure (217). These findings indicate that the inflammatory environment and lymphatic clearance could compromise the stability of Tregs and restrict their efficacy in the context of CAV.

Recent advancements in autologous Treg sources and preparation technologies have led to significant breakthroughs in the prevention and treatment of CAV. Bernaldo-de-Quirós et al. produced high-purity and high-activity thymus-derived Tregs (thyTregs) from thymus tissue excised during pediatric cardiac surgery, yielding a substantial quantity of Tregs with a stable phenotype and robust inhibitory function. This scheme has received approval to proceed to the clinical research phase (218). After the initial instance of infant heart transplantation, no adverse reactions were noted after two years of follow-up following the infusion of autologous thyTregs. The proportion of peripheral Tregs was sustained over an extended period, thereby providing initial confirmation of the safety of this therapy and its potential for long-term immune homeostasis reconstruction (219). Furthermore, adjuvant immunomodulatory therapies could indirectly improve Treg homeostasis. Extracorporeal photopheresis (ECP) significantly increases the proportion and inhibitory function of peripheral CD4^+^Foxp3^+^ Tregs in heart transplant recipients, which improves immune tolerance post-transplantation and potentially delays the progression of CAV (220).

Research on various organ transplants has corroborated the viability of autologous Treg therapy. In the context of kidney transplantation, the reinfusion of autologous polyclonal Tregs could lead to a stable increase in peripheral Treg populations, decrease the incidence of acute rejection, and allow lowering of recipients’ immunosuppressant requirements (221). Additionally, the delayed infusion of autologous Tregs following lymph node clearance contributes to the restoration of Treg-dominated immune balance, offering insights into the timing of immune intervention for the treatment of CAV (222). In summary, autologous Treg therapy represents a novel approach for cell immunotherapy to prevent and treat CAV through the restoration of immune tolerance, the mitigation of chronic inflammation, and the inhibition of vascular remodeling, providing a clinical basis for future precision immune regulation.

### 6.2 Strategies for Treg expansion *in vitro*

Tregs are constrained *in vivo*, and their large-scale expansion *in vitro* presents a significant technical challenge for clinical application (223). Researchers are actively developing efficient and stable systems to increase the expansion capability, purity, and functional stability of Tregs. Hoffmann et al. reported that a culture strategy utilizing artificial antigen-presenting cells in conjunction with high-dose IL-2, which facilitated the *in vitro* amplification of human CD4^+^CD25^high^ Tregs by up to 40, 000 times. The resulting amplified cells exhibited a stable polyclonal phenotype and retained inhibitory activity (224). Furthermore, to facilitate the rapid evaluation of Treg function in clinical settings, Canavan et al. developed a flow cytometry detection method that requires only 7 hours, thereby offering a practical tool for monitoring functional Tregs (225). To increase Treg purity, certain studies have implemented a separation process adhering to good manufacturing practice (GMP) standards, resulting in a Treg population characterized by high purity, robust inhibitory capacity, and significant amplification potential through the elimination of CD127-positive cells (226).

Consequently, to advance the clinical application of Treg therapy, researchers have systematically proposed and refined a series of production processes that adhere to GMP standards. An efficient isolation and amplification system has been established for thymus-derived Tregs from children, and this system has demonstrated good therapeutic potential and functional stability (218). A separate investigation demonstrated that the combination of a TNFR2 agonist and rapamycin for the expansion of low-purity Tregs yields a homogeneous and functionally stable inhibitory cell population that exhibit a sustained immunomodulatory function *in vitro* over an extended period (227). The application of a CD28 superagonist antibody markedly increased the *in vitro* expansion efficiency of Tregs, with the expanded cells retaining characteristic phenotypes and inhibitory functions (228), thus offering a viable approach for the generation of clinical-grade Tregs. Recent study has indicated that IL-6 and TNF-α could synergistically promote the extensive proliferation of Tregs *in vitro* while preserving their lineage stability and regulatory function (229), suggesting new avenues for cytokine-regulated expansion protocols. On the basis of the above amplification strategy, Romano et al. advanced a GMP-compatible amplification process for thymus-derived Tregs that exhibited efficient cell amplification and functional stability and confirmed the significant *in vitro* inhibitory capacity of the amplified Tregs (230). These systematic studies have not only improved the amplification efficiency, purity, and functional reliability of Tregs but have also established a solid foundation for the clinical transformation of this type of therapy in the areas of tolerance induction in transplantation and autoimmune disease treatment (231, 232). Current strategies for *in vitro* Treg expansion are summarized in **Table 2**.

**Table 2:** Strategies for Treg expansion *in vitro*.

Amplification strategy	Fundamental mechanisms	Advantages	Disadvantages/challenges	References
IL-2 + APCs	Mimicking physiological TCR/IL-2 signals	High amplification and maintenance of polyclonal nature	APC-dependent; activate Teffs; high cost	(224)
Rapamycin	mTOR inhibition; selectively promotes Treg proliferation	Enhance the quality and stability of Tregs while suppressing Teffs	Modulate Treg functionality; dosage optimization is required	(108, 230)
ATRA + Rapamycin	Synergistically promote Treg expansion and stability	Augment the epigenetic stability of FOXP3 and enhance its inhibitory efficacy	Complexity of drug combinations; potential side effects	(108)
TNFR2 agonist + Rapamycin	Enhance Treg proliferation and maintain function	Efficiently expand low-purity Tregs and reduce pro-inflammatory cytokines	Clinical safety to be verified	(227)
IL-6 + TNF-α	Enhance Treg proliferative response and maintain lineage stability	Provide new cytokine combination amplification method	Mechanism requires additional elucidation; a risk of pro-inflammatory responses	(229)
Orthogonal IL-2 system	Engineered IL-2 and its receptor to specifically activate Tregs	Accurately expand Tregs and avoid non-specific activation of Teffs	Clinical transformation is intricate, as it is still in the early phases of research and development	Prospective derived from (247)

## 7 Challenges and prospects

The growing understanding of Treg biology in CAV has laid a foundation for developing Treg-based therapeutic strategies. However, translating these fundamental insights into effective clinical therapies is confronted by substantial barriers including manufacturing complexity, Treg instability, insufficient *in vivo* persistence and the limited availability of biomarkers for monitoring therapeutic efficacy (151, 152). Addressing these challenges while simultaneously pursuing innovative approaches to harness Treg-mediated immune regulation will be critical for moving toward the goal of achieving immune tolerance in heart transplantation.

### 7.1 Challenges in clinical translation

Clinical translation of Treg-based therapy for CAV is constrained by interconnected barriers, from Treg manufacturing and phenotypic stability to clinical trial design and the challenges imposed by the allograft microenvironment. The primary issue is the maintenance of Treg phenotypic and functional stability of adoptively transferred cells. Effector memory Tregs have been shown to display instability, raising concerns about conversion to pro-inflammatory effectors in the CAV microenvironment (233). Meanwhile, a related obstacle involves the scalable manufacture of Tregs under GMP-compliant conditions. Large-scale expansion protocols that preserve Treg potency remain demanding, particularly with respect to sustaining Foxp3 expression, suppressive capacity, and lineage fidelity throughout the production process (223, 224). The design of clinical trials for Treg therapy in CAV also presents unique considerations, as demonstrated by the ATT-Heart trial, which highlighted the need for rigorous dose-escalation designs, appropriate patient selection, and long-term immunological monitoring in pediatric heart transplant recipients (234). In addition, the chronic inflammatory milieu of CAV could compromise the persistence and function of infused Tregs, necessitating repeated administration or combination with selective immunosuppressive agents that do not impair Treg function (151, 152, 217). Besides, early-phase clinical trials have demonstrated that Treg therapy could facilitate immunosuppression reduction and weaning, emphasizing the need for reliable monitoring tools to guide such protocols (221, 222).

### 7.2 Prospects associated with clinical tolerance

Chimeric antigen receptor (CAR)-Treg technology represents a promising strategy for achieving donor-specific tolerance in CAV (235). By redirecting Treg specificity toward donor HLA molecules, CAR-Tregs could deliver targeted suppression at the graft site while sparing systemic immunity (236). Supraphysiological expression of FOXP3 in human CAR-Tregs has been shown to improve their stability, efficacy, and safety, addressing important questions regarding lineage fidelity and functional persistence (236, 237). Furthermore, HLA matching or clustered regularly interspaced short palindromic repeats (CRISPR)-mediated editing of HLA class I/II molecules has enabled the development of allogeneic Treg products, potentially overcoming the scalability limitations of autologous Treg manufacturing (237). Recent studies have further highlighted the potential of CAR-Treg approaches for achieving durable allograft tolerance in transplantation, providing a conceptual framework for developing tolerance-inducing strategies applicable to CAV (238). Besides, The generation of tacrolimus-resistant Tregs offers a complementary strategy to enable concurrent Treg therapy and conventional CNI-based immunosuppression without compromising Treg function (239). These engineering advances bring antigen-specific Treg therapy closer to clinical application for CAV prevention.

The feasibility of Treg-mediated tolerance in transplantation has been widely confirmed by both fundamental research and clinical evidence. Todo et al. demonstrated in a pilot clinical trial that Treg-cell therapy facilitated operational tolerance in living donor liver transplant recipients, with successful immunosuppressants withdrawal in a proportion of patients (240). The observation in liver transplantation, though requiring direct validation in heart transplant recipients, provides a valuable foundation for the development of tolerance-inducing strategies specifically designed for CAV. Besides, Ren et al. showed that oxymatrine, a natural compound with immunomodulatory properties, attenuates chronic allograft rejection by restoring Treg frequency and function while inhibiting fibrotic remodeling, suggesting that pharmacological agents capable of bolstering Treg activity may serve as adjuncts to cell-based therapies for CAV prevention (241). Che et al. also indicated that targeting high endothelial venules with a specialized antibody-drug conjugate promotes long-term cardiac allograft acceptance, and this protective effect is associated with enhanced intragraft Treg enrichment, highlighting the importance of Treg generation at the graft site in sustaining immune homeostasis and preventing CAV (242). Nevertheless, substantial preclinical investigations remain warranted to translate these mechanistic findings into viable strategies for inducing clinical transplant tolerance.

The integration of multi-omics technologies, including spatial transcriptomics and proteomics, as recently applied to the analysis of CAV arterial lesions (8), will be instrumental in elucidating Treg behavior within the CAV lesion microenvironment and identifying clinically applicable biomarkers of tolerance. Single-cell transcriptomic studies have already begun to reveal the heterogeneity of intragraft Tregs, identifying Amphiregulin (Areg)-expressing Treg subsets with tissue repair potential whose functional integrity is compromised during chronic rejection (183, 235). These tools could facilitate the development of personalized Treg therapy protocols and enable real-time monitoring of graft immune status, ultimately aiding the induction of clinical transplant tolerance.

### 7.3 Clinical endpoints for tolerance trials

The Fourth International Workshop on Clinical Transplant Tolerance summarized presentations regarding the status of clinical trials designed to minimize or withdraw immunosuppressive drugs in solid organ transplantation with no subsequent evidence of rejection (243). Sustained graft function after immunosuppression withdrawal defines the clinical endpoint for these tolerance-induction trials, an approach that could be applied to heart transplantation. Meanwhile, Serial IVUS measurements provide quantifiable surrogate endpoints in heart transplantation. Kim et al. reported in a 10-year follow-up study that baseline maximal intimal thickness (MIT) and 1-year changes in MIT by IVUS were associated with long-term cardiovascular outcomes in heart transplant recipients, supporting IVUS-derived parameters as surrogate endpoints (244). Okada et al. demonstrated that attenuated-signal plaque progression detected by IVUS predicted long-term mortality after heart transplantation (245). The EVOLVD trial used coronary artery intima thickness measured by IVUS as the primary endpoint, illustrating how IVUS-based surrogate endpoints are implemented in clinical trial designs (246). Future trials should integrate clinical, surrogate, and even biomarker-based endpoints to evaluate whether Treg-based strategies achieve tolerance in heart transplantation.

**Table 3** provides a summary of evidence derived from human studies, animal models, and *in vitro* experiments throughout this review, with observations classified according to the Technology Readiness Level (TRL) framework to indicate their translational maturity, thereby facilitating an integrated assessment of the evidence for Treg-targeted therapies in CAV.

**Table 3:** TRL levels of evidence for Treg-associated observations in CAV.

Topics	Models (types)	Summaries of evidence	TRL levels	References
Alloimmunity	Human (Clinical)	TSP-1/AIF-1 upregulation linked to CAV	TRL 1	(63, 64)
	Animal (Preclinical)	NK cells participate in rejection via Fc receptors and antibodies/IL-6		(45, 47, 197)
		CCL21/CXCR3 is a therapeutic target for CAV	TRL 3	(38)
		Noncanonical NF-κB involved in CAV	TRL 1	(6)
	*In vitro* (Mechanistic)	NF-κB regulates endothelial inflammation		(36, 40)
Inflammatory microenvironment	Human (Clinical)	Plasma CRP/VCAM-1/neopterin associated with CAV	TRL 1	(196)
	Animal (Preclinical)	ATG and IL-6 inhibit pro-Tregs and resist refractory rejection	TRL 3	(53)
	Animal (Preclinical)	MEK1/2-PKM2 regulates macrophage proinflammatory phenotype and glycolysis		(43)
VSMC phenotype switching	Animal (Preclinical)	Anti-vimentin autoimmunity, neutrophils, and AXL/PDGF/CYP2C pathways promote VSMC proliferation; CXCR4 blockade and anti-LFA-1 attenuate	TRL 1	(60, 65–67, 69, 72, 74)
		Egr-1/p300/CBP/Ets-2 regulate VSMC proliferation and plasticity		(62, 70, 71)
		Platelet microvesicles promote VSMC dedifferentiation via Src/Lamtor1/mTORC1		(61)
	*In vitro* (Mechanistic)	CMV/MMP-14/allogeneic lymphocyte stimulation affect VSMC		(58, 68, 73)
Diagnosis	Human (Clinical)	^13^N-ammonia PET assesses MBFR with prognostic value	TRL 6	(2)
		Novel protein markers and hs-cTn/NT-proBNP associated with CAV	TRL 1	(91, 92)
		iCAV-Pr and machine learning models predict allograft rejection	TRL 6	(94, 95)
Factors maintaining Treg phenotype	Animal (Preclinical)	Mettl14-m6A modification critical for Treg function	TRL 1	(136)
		miR-146a regulates Tregs via IFN-γ/STAT1, affecting transplant outcomes		(137)
		Membrane-bound IL-2 improves CAR-Treg expansion/survival	TRL 3	(112)
Factors maintaining Treg phenotype	*In vitro* (Mechanistic)	TGF-β sustains iTreg stability; disruption of TGF-β/SKI exerts adverse impacts, which could be counteracted by ARKADIA	TRL 1	(115, 116, 118)
		Retinoic acid and rapamycin synergistically expand natural Tregs	TRL 3	(108)
Targeting Tregs to prolong graft survival	Animal (Preclinical)	Treg infusion could achieve mixed chimerism and inhibit chronic rejection	TRL 3	(18, 19)
		IL-2/anti-IL-2 complexes or low-dose IL-2 expands Tregs and prevents CAV		(20, 147)
		IL-35 promotes Treg stability and protects grafts		(149)
Treg homeostasis dysregulation	Human (Clinical)	Th1/Treg ratio and PD-1 inhibitors associated with CAV	TRL 1	(154, 162)
	Animal (Preclinical)	Treg depletion promotes NK-mediated CAV		(215)
PD-1/PD-L1 pathway	Animal (Preclinical)	PD-1/PD-L1 augments Tregs through multiple mechanisms; PD-1/PD-L1 deficiency aggravates allograft rejection and exacerbates CAV	TRL 1	(174–176, 178, 179)
IL-33 pathway	Animal (Preclinical)	IL-33/ST2 regulates macrophages and expands Tregs; ST2 deficiency yields the opposite effect and aggravates CAV	TRL 1	(185, 186, 188, 189)
IL-6 pathway	Human (Clinical)	Plasma IL-6 correlates with intimal thickening/inflammation	TRL 1	(196)
	Animal (Preclinical)	IL-6 inhibition promotes Tregs; anti-IL-6 prolongs survival; Jagged2 increases IL-6 to inhibit Tregs; donor IL-6 promotes intimal hyperplasia; IL-6 promotes hyperlipidemia accelerating rejection	TRL 3	(52, 53, 191, 192, 194)
CTLA-4 pathway	Animal (Preclinical)	Low-dose CTLA-4-Ig is Treg-dependent; rapamycin facilitates Tfh activation, causing antibody-mediated vascular injury; EPO synergizes with CTLA-4-Ig to expand Tregs; hyperlipidemia induces Treg dysfunction and CTLA-4-Ig tolerance resistance; costimulatory blockade sustains cardiac allograft tolerance	TRL 3	(200–205)
FAO pathway	*In vitro* (Mechanistic)	PPARγ upregulates FAO via CD36/CPT1 to enhance Tregs	TRL 1	(209)

## 8 Conclusion

CAV remains the principal obstacle to long-term survival after heart transplantation, driven by a complex interplay of alloimmune responses, inflammatory signaling, and vascular remodeling. Tregs are essential for maintaining graft immune homeostasis through their capacity to suppress effector responses and promote tolerance. Within the CAV microenvironment, however, Treg function is progressively undermined at multiple levels, including Foxp3 instability, metabolic compromise, defective trafficking, and checkpoint dysregulation. Moreover, signaling pathways such as PD-1/PD-L1, IL-33/ST2, IL-6, CTLA-4, the liver-derived PCSK9/CD36, and metabolic pathways including FAO form cross-organ regulatory networks that govern Treg recruitment, function, and metabolism while connecting hepatic metabolic status to the cardiac graft microenvironment through donor heart-liver crosstalk.

Despite the above-documented findings and analyses, several issues remain unresolved. The dynamics of checkpoint molecule expression on intragraft Tregs during CAV progression require further characterization, and robust biomarkers for monitoring Treg therapy efficacy have yet to be established. The optimal protocols for Treg delivery and the maintenance of their functional stability within the inflammatory CAV microenvironment also require further investigation. Future research priorities should include the development of standardized GMP-compliant Treg manufacturing protocols, the integration of multi-omics technologies and CAR-Treg engineering, and the design of clinical trials incorporating tolerance-relevant surrogate endpoints such as IVUS-derived parameters. Addressing these issues will be essential for translating Treg-based strategies into durable clinical tolerance in heart transplantation.

## Glossary

ABCB5: ATP-binding cassette sub-family B member 5
AIF-1: Allograft inflammatory factor-1
AIMs: Allograft-infiltrating macrophages
AMR: Antibody-mediated rejection
Areg: Amphiregulin
ATG: Anti-thymocyte globulin
ATRA: All-trans retinoic acid
AUROC: Area under the receiver operating curve
BNP: Brain natriuretic peptide
CAR-Tregs: Chimeric antigen receptor-engineered regulatory T cells
CAV: Cardiac allograft vasculopathy
CBP: CREB-binding protein
CCL21: C-C motif chemokine ligand 21
CCR5: C-C chemokine receptor type 5
CMV: Cytomegalovirus
CNIs: Calcineurin inhibitors
CPT1: Carnitine palmitoyltransferase 1
CRAC: Ca^2+^ release-activated Ca^2+^
CRISPR: Clustered regularly interspaced short palindromic repeats
CTLA-4-Ig: Cytotoxic T lymphocyte associated protein 4-immunoglobulin fusion protein
CXCR3: C-X-C motif chemokine receptor 3
CYP2C: Cytochrome P450 2C
darTregs: Donor antigen-alloreactive regulatory T cells
dd-cfDNA: Donor-derived cell-free DNA
DIRCs: Dermal immunoregulatory cells
DSAs: Donor-specific antibodies
ECP: Extracorporeal photopheresis
Egr-1: Early growth response factor-1
EMB: Endomyocardial biopsy
EPO: Erythropoietin
Ets-2: Erythroblast transformation-specific 2
ex-Tregs: CD3^+^CD4^+^CD16^+^CD56^+^CD25^low^Foxp3^low^ regulatory T cells
FAO: Fatty acid oxidation
GLS: Global longitudinal strain
GMP: Good manufacturing practice
gp130: Glycoprotein 130
HLA: Human leukocyte antigen
ICAM-1: Intercellular adhesion molecule-1
ICOS: Inducible T-cell costimulator
iTregs: Induced regulatory T cells
IVUS: Intravascular ultrasound
m6A: N6-methyladenosine
MAC: Membrane attack complex
MBFR: Myocardial blood flow reserve
MDSCs: Myeloid-derived suppressor cells
MEK1/2: Mitogen-activated protein kinase kinase 1/2
Mettl14: Methyltransferase-like 14
MIT: Maximal intimal thickness
MMP-14: Matrix metalloproteinase-14
mTOR: Mammalian target of rapamycin
Nrp-1: Neuropilin-1
NT-proBNP: N-terminal pro-B-type natriuretic peptide
nTregs: Natural regulatory T cells
OXPHOS: Oxidative phosphorylation
PARP-1: Poly(ADP-ribose) polymerase 1
PCSK9: Proprotein convertase subtilisin/kexin type 9
PDGF: Platelet-derived growth factor
PF: Platelet factor
PKM2: Pyruvate kinase M2
PRAs: Panel reactive antibodies
pTregs: Peripheral-induced regulatory T cells
Rag: Recombination activating gene
RORγt: Retinoic acid related orphan receptor γt
scRNA-seq: Single-cell RNA sequencing
SKI: SKI proto-oncogene
SOCS1: Suppressor of cytokine signaling 1
ST2L: A longer isoform of suppression of tumorigenicity 2
STAT: Signal transducer and activator of transcription
Teffs: Effector T cells
Tfam: Mitochondrial transcription factor A
Tfhs: T follicular helper cells
TGF-β: transforming growth factor-β
Th1: T helper 1 cells
Th17: T helper 17 cells
Th2: T helper 2 cells
thyTregs/tTregs: Thymus-derived regulatory T cells
TIGIT: T cell immunoreceptor with Ig and ITIM domains
TNFR2: Tumor necrosis factor receptor 2
Tregs: Regulatory T cells
TRL: Technology Readiness Level
TSP-1: Thrombospondin-1
VCAM-1: Vascular cell adhesion molecule-1
VEGF: Vascular endothelial growth factor
VSMCs: Vascular smooth muscle cells
